# All‐inside single‐bundle and modified double‐bundle anterior cruciate ligament reconstruction techniques guarantee stability and similar clinical results at over 5 year follow‐up

**DOI:** 10.1002/jeo2.70100

**Published:** 2025-02-27

**Authors:** Lorenzo Moretti, Raffaele Garofalo, Giuseppe Danilo Cassano, Roberto Calbi, Francesco Fiore, Giuseppe Solarino

**Affiliations:** ^1^ Department of Translational Biomedicine and Neuroscience (DiBraiN), Orthopaedic and Trauma Unit, AOUC Policlinico di Bari University of Bari “Aldo Moro” Bari Italy; ^2^ Department of Orthopaedics and Traumatology Ente Ecclesiastico Ospedale “F. Miulli” Acquaviva delle Fonti Bari Italy

**Keywords:** ACL, anterior cruciate ligament reconstruction, double bundle, knee, single bundle

## Abstract

**Purpose:**

The aim of the present study was to compare clinical and radiological outcomes between the all‐inside single bundle (SB) and a modified double‐bundle (DB) anterior cruciate ligament reconstructions (ACLR) at over 5‐year follow‐up.

**Methods:**

This is an observational, retrospective comparative, two‐centre study. Clinical outcomes were evaluated using Lysholm and International Knee Documentation Committee (IKDC) scores, and anterior tibial translation (ATT) was assessed using the KT‐1000 arthrometer. Knee x‐ray images were recorded, classified according to the KL grading and compared with radiographs of the same patient before surgery. Inclusion criteria were patients undergoing ACLR, age between 18 and 45 years and negative knee history of major traumatic events after surgery. Exclusion criteria were congenital laxity, combined multiple knee ligament injuries, patients undergoing ACL revision surgery, history of infection, lower limb coronal axial deviation >5°, patients undergoing lateral extra‐articular tenodesis or anterolateral ligament reconstruction, patients with chondral damage Outerbridge grade >2, patients with meniscal tears undergoing subtotal meniscectomy or meniscal repair and patients with knee OA Kellgren–Lawrance (KL) grade >3.

**Results:**

One hundred and fifty‐two patients were included in the study. Patients were divided into two groups according to surgical technique: Group A—ACLR with all‐inside technique, and Group B—ACLR with modified DB technique. There were no statistical differences between groups for age, side, gender or time since surgery.

There were no statistically significant differences between groups for Lysholm scores (*p* = 0.43), IKDC (*p* = 0.88), ATT (*p* = 0.105) and KL grade (*p* = 0.93 before surgery, 0.99 at the fu). KL grade increased significantly since pre‐op.

**Conclusions:**

Our data show significant improvements in all clinical outcome measures, along with excellent KT‐1000 arthrometer values and low clinical failure rates for both the SB all‐inside and modified DB techniques at a mean follow‐up of over 6 years. There were no significant differences in arthritic progression according to KL grade between groups.

**Level of Evidence:**

Level III.

**Abbreviations**
ACL: anterior cruciate ligament
ACLR: anterior cruciate ligament reconstruction
AMB: anteromedial bundle
ATT: anterior tibial translation
DB: double bundle
IKDC: International Knee Documentation Committee
KL: Kellgren–Lawrance
NA: nonanatomical
OA: osteoarthritis
PLB: posterolateral bundle
SB: single bundle

## INTRODUCTION

Anterior cruciate ligament reconstruction (ACLR) is a widely used treatment for active individuals with ACL injuries, aiming to restore stability and function of the knee joint. There are different graft choices and different techniques for ACLR [10, 13, 14, 15, 16, 17, 18, 19, 20, 21, 22, 23, 24, 25, 26, 27, 28, 29, 30]. These different techniques can affect the ‘ligamentization’ process and the final outcomes [9, 25]. In recent years, both single‐bundle (SB) and double‐bundle (DB) techniques have been widely used in ACLR. On the other hand, there was no consensus on whether the DB technique was better than the SB technique [38]. It is well known that the anteromedial bundle (AMB) and the posterolateral bundle (PLB) are the two functional bundles that comprise the ACL. Within the joint, these two grafts cross each other and work independently at various knee angles. Theoretically, the PLB may also limit anterior tibial loads and a combined rotational loading at lower flexion angles, while the AMB can prevent anterior tibial translation (ATT) at higher flexion angles [11]. According to several biomechanical studies [14, 20, 21, 22, 23, 24, 25, 26, 27, 28, 29, 30, 31], the DB technique has the potential to repair both the AMB and the PLB, which could result in knee stability and kinematics that are more similar to the natural knee than those produced by the SB technique in ACLR. However, additional biomechanical research [21, 22] has shown that the DB reconstruction may not provide significant additional benefits over the SB reconstruction.

According to a meta‐analysis of randomised controlled trials, the DB technique was not superior to the SB technique in autologous ACL reconstruction in terms of knee stability, clinical function and graft failure rate at 5‐year follow‐up [6].

ACL injury predisposes knees to osteoarthritis (OA), while ACLR surgery has a role in reducing the risk of developing degenerative changes at 10 years [2]. However, the role of these ACLR techniques in delaying or preventing the development of joint arthritis is also controversial, and a recent meta‐analysis showed that the DB technique was no more effective than the SB technique in preventing the progression of OA at mid‐term follow‐up [38].

There are different ACLR DB techniques, with different positions and numbers of tunnels or different fixation devices [12]. The position of the tunnels is essential to restore the native kinematics of the knee and to ensure better rotational stability [27, 28, 29, 30, 31, 32].

Zantop et al. in a controlled laboratory study demonstrated that DB ACLR using the anatomical PLB tunnel position restores the intact knee kinematics. A nonanatomical (NA) PLB position results in rotatory instability [36]. However, other authors have shown that NA DB ACLR can control anterior–posterior laxity and the pivot‐shift phenomenon as well as anatomic DB ACLR [35].

The all‐inside technique is one of the ACLR SB techniques and has some advantages, including a closed tunnel with less bone displacement which may be helpful if subsequent revision reconstruction, the use of the semitendinosus tendon as the only graft, partial violation of extra‐articular cortices and periosteum, double cortical suspensory fixation and smaller incisions [3].

Furthermore, this technique easily allows the dedicated guides to position the tunnels anatomically or between the 9 and 10 o'clock position for the right knee and the 14 and 15 o'clock position for the left knee, respectively [16].

There are few studies in the literature comparing the outcomes between SB all‐inside and DB ACLR techniques but with short‐term follow‐up and small sample sizes [26]. It would be useful to have a study with a larger sample size and longer‐term follow‐up, so it was decided to carry out this study.

The aim of the present study was to compare clinical and radiological outcomes between the all‐inside SB and a modified DB ACLR at over 5‐year follow‐up. The hypothesis of the study is that there is no significant difference between the two techniques.

## MATERIALS AND METHODS

### Study design and sample

This is an observational, retrospective comparative, two‐centre study. The study was approved by the ethical committee, all patients gave informed consent prior to enrolment.

Inclusion criteria were patients undergoing ACLR, age between 18 and 45 years and negative knee history of major traumatic events after surgery. Exclusion criteria were congenital laxity, combined multiple knee ligament injuries, patients undergoing ACL revision surgery or new arthroscopy for meniscus or cartilage surgery, history of infection, lower limb coronal axial deviation >5°, patients undergoing lateral extra‐articular tenodesis or anterolateral ligament reconstruction, patients with chondral damage Outerbridge grade >2, patients with meniscal tears undergoing subtotal meniscectomy or meniscal repair and patients with knee OA Kellgren–Lawrance (KL) grade >3.

Three hundred and sixty‐five patients were identified in the database from 2015. About 213 were excluded (190 did not meet inclusion and exclusion criteria, and 23 refused to participate in the study), leaving 152 patients included. They were evaluated between April and September 2023. For each patient, the following data were recorded: age, sex, side of injury, time since surgery (rounding up from the sixth month) and x‐ray images.

**Figure 1:**
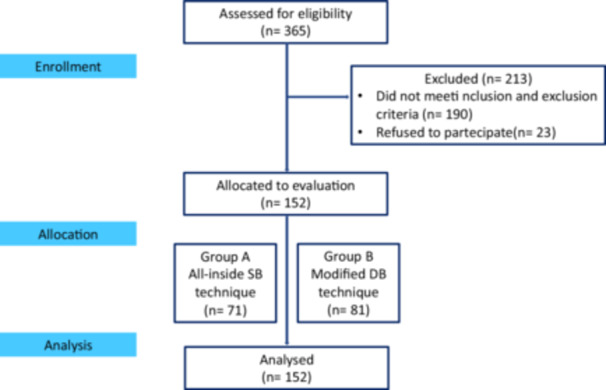
Study phases.

The patients were divided into two groups according to surgical technique: Group A—ACLR with all‐inside technique (ACLR performed by a single surgeon with a high volume of ACLR with all‐inside technique), and Group B—ACLR with modified DB technique (ACLR performed by a single surgeon with a high volume of ACLR with modified DB technique). The flowchart of patient selection is shown in Figure 1.

### Clinical evaluation

Anterior knee laxity was assessed by two senior fellows at our outpatient clinic using the KT‐1000 arthrometer. An 89‐N anterior tibial load, at 20° of knee flexion, was applied. At least three measurements for each knee were performed and the median value was registered. The ATT was expressed in millimetres. ATT value > 5 mm is an indicator for ACLR failure as described in a recent study [10]. After clinical evaluation, as secondary endpoint Lysholm questionnaire [23] and the International Knee Documentation Committee (IKDC) [18] questionnaire have been used to evaluate Quality of Life and subjective outcomes.

As a tertiary endpoint knee x‐rays (antero‐posterior and latero‐lateral projections) were recorded, classified according to the KL grading [19] and compared with radiographs of the same patient before surgery.

### Surgical technique

Surgery is performed under spinal anaesthesia, with the patient placed in a supine position on the operating table. After pneumatic tourniquet positioning and preparation of the leg, we created the arthroscopic portals.

In group A, ACLR all‐inside technique with Quadrupled Semitendinosus graft (ST4) was performed [4]. A 110° femoral aimer (Femoral curved ACL Marking Hook, Arthrex©) and a 55° tibial aimer (Tibial ACL Marking Hook, Arthrex©) were pointed to the anatomical ACL footprints at the 10 o'clock position under direct arthroscopic view. The retrograde femoral and tibial half tunnels using FlipCutter® II Drill (Arthrex©) were created; it measured about 2.5 cm. The graft was fixed with the knee in extension with a cortical suspension system (Tight‐Rope, Arthrex©) on both femoral and tibial sides.

**Figure 2:**
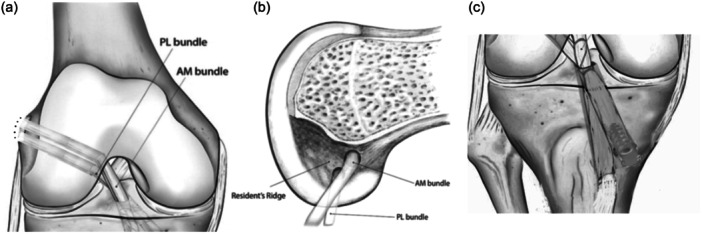
Femoral and tibial tunnels. (a) Coronal plane view of the femoral tunnels; (b) Sagittal plane view of the femoral tunnels; (c) Coronal plane view of the tibial tunnels.

**Figure 3:**
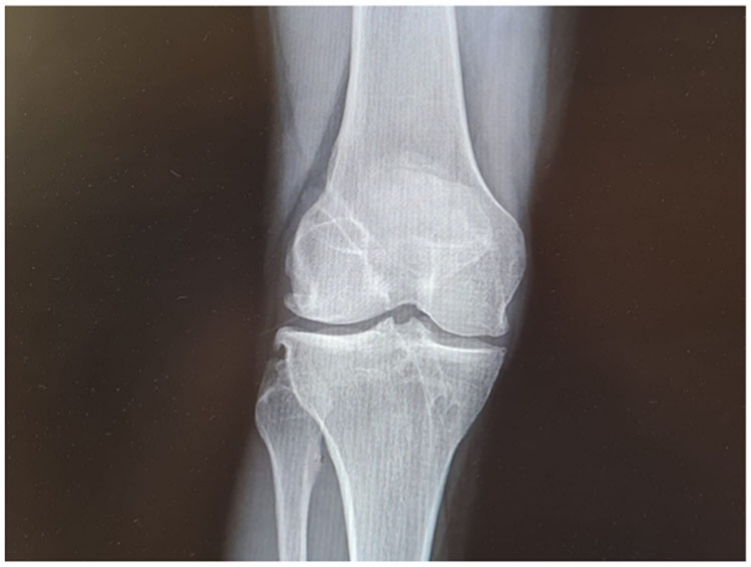
Coronal x‐ray image of a group B patient at final follow‐up.

In group B, a modified over‐the‐top DB technique was performed using the gracilis and semitendinosus tendons [33]. They were harvested leaving the tibial insertion intact, dissected and sutured at the ends and sutured together. Using an anteromedial portal technique and with a 6 mm drill, two complete independent femoral tunnels were created through the anteromedial portal with the knee held in 110 degrees of flexion and in four leg position, starting at the level of the footprint of the AM and PL bundles. A direct drill guide (Acufex, Smith & Nephew) was set at 55° for the PL tibial tunnel and 45° for the AM tibial tunnel and a 6 mm drill was used. Nitinol wires were used as carriers. The tendons were passed inside the posterior tibial tunnel and then inside the most posterior femoral tunnel. They were passed outside the cortical bone and inside the second femoral tunnel. Finally, they were passed back inside the tibial tunnel and with the knee in extension, and in the posterior drawer, the system was locked using an interference screw (Biosure, Smith & Nephew). The technique is illustrated in Figure 2; an x‐ray image is shown in Figure 3.

### Statistical analysis

Data were collected and analysed using Microsoft Excel and SPSS IBM. Categorical variables were presented as numbers or percentages. Continuous variables were presented as mean and standard deviation (SD). All variables were tested for normality using the Shapiro–Wilk test. The Mann–Whitney U test was used when normality was rejected (ATT, Lyshom, IKDC and OA progression in the two groups).

The *χ* ^2^ test was used for group comparisons of two categorical variables (differences in gender and side).

A *p *< 0.05 was considered statistically significant. Data presented in this study are available on request from the corresponding author.

Based on previously published data [1], a Power analysis, with a power of 80% and an α of 0.05, showed that a sample size of 28 patients in each group was needed to detect a 1‐mm difference compared with contralateral side with a 1.3‐mm SD in KT‐1000 measurements.

## RESULTS

One hundred and fifty‐two subjects were enrolled in this study and divided into two groups: group A was composed of 71 patients (27.25 ± 6.51 mean age‐years, 53 male, 6.39 ± 1.11 mean time evaluation after surgery‐years) and group B was composed of 81 patients (27.43 ± 6.55 mean age‐years, 62 male, mean time evaluation after surgery‐6.32 ± 0.99 years).

We compared the study and control group at recruitment. The main demographic characteristics are described in Table 1. No statistical differences emerged between groups for age, side, gender, time from surgery and time from injury to surgery.

**Table 1:** Baseline evaluation of study participants.

Preoperative features	Group A	Group B	*p* Value
Number of patients	71	81	‐
Age	27.25 ± 6.51	27.43 ± 6.55	0.94
Side (right)	37	36	0.34
Gender (male)	53	62	0.78
Time from surgery (years)	6.39 ± 1.11	6.32 ± 0.99	0.28
Time from injury to surgery (months)	3.89 ± 1.87	4.01 ± 2.05	0.79

*Note*: No statistical differences emerged between groups.

The ATT expressed in millimetres, Lysholm score and IKDC score were calculated and are shown in Table 2. The Shapiro–Wilk test showed a nonnormal distribution for ATT values, Lysholm score values and IKDC values, so the Mann–Whitney *U* test was used to compare values between groups.

**Table 2:** Differences in anterior knee laxity, Lysholm score and IKDC score between groups at the follow‐up.

	Group A	Group B	*p* Value
ATT (mm)	2.8 ± 1.79	2.77 ± 2.31	0.105
Lysholm	92.01 ± 7.29	91.47 ± 6.85	0.43
IKDC (%)	92.90 ± 4.84	93 ± 5.63	0.88

*Note*: Data are presented as mean ± standard deviation. No statistical differences emerged between groups.

Abbreviations: ATT, anterior tibial translation; IKDC, International Knee Documentation Committee.

Patients were divided according to KL grading before and at the evaluation time as shown in Table 3. The Mann–Whitney *U* test was used to compare values between groups. Before and after surgery, no statistical differences emerged between groups (*p* values were, respectively, 0.93 and 0.99).

**Table 3:** Difference in OA between groups at baseline and follow‐up, by number of patients according to KL grading.

KL grade	Group A		Group B	
	Pre	Follow‐up	Pre	Follow‐up
0	27 (38%)	6 (8.5%)	31 (38%)	7 (8.6%)
I	37 (52.1%)	49 (69%)	41 (51%)	56 (69.1%)
II	6 (8.5%)	14 (19.7%)	7 (8.6%)	14 (17.3%)
III	1 (1.4%)	2 (2.8%)	2 (2.4%)	4 (5%)

Abbreviations: KL, Kellgren–Lawrance; OA, osteoarthritis.

In both groups A and B emerged a statistically significant difference (*p* < 0.05) according to the evolution of the KL grade between presurgery and follow‐up.

## DISCUSSION

The main findings of this study are that there are no differences in ATT, IKDC and Lysholm scores between the all‐inside SB technique and the modified DB technique. In the literature, only a study compares the all‐inside technique versus the DB technique, but it has a limited sample size and short‐term follow**‐**up. The authors do not show any differences between the groups when considering IKDC and Lysholm scores. IKDC and Lysholm scores reported in our series are slightly better than those obtained and described by R. Novriansyah and colleagues [26]. There are no other comparative studies. In addition, our work considers a variation of the DB technique with double femoral tunnel with an extracortical graft passage. Both are anatomical ACLR techniques, but only in the all‐inside there is a bone spearing, while in the DB two complete tunnels with a 6 mm diameter are performed on both the femoral and tibial sides. As is well known, the all‐inside technique uses only the semitendinosus tendon, whereas the DB technique also uses the gracilis tendon.

The technique used in group B allows the preservation of the hamstring tendon insertion, which could improve the ‘neoligamentization’ process [34].

Several studies have compared SB techniques with DB techniques: Chen et al. [5] performed a meta‐analysis of randomised controlled trials comparing traditional SB and DB techniques and found no significant difference in IKDC scores. Chen et al. [7] compared the clinical impact of individualised anatomical SB and DB and showed no significant difference between the two groups in terms of the IKDC, Lysholm and Tegner scores. Another study by Zhang et al. [37] also showed no statistically significant differences between SB and DB ACL reconstructions on Tegner and Lysholm scores at 24 months.

There are several studies evaluating the outcomes of the all‐inside technique [15] and DB techniques (anatomical and NA) [29] with excellent results. Monaco et al. in a 2‐year follow‐up study of ACLR using the all‐inside technique, found similar results to those shown for group A in our study, with a mean IKDC score of 91 (87.4–100), a mean Lysholm score of 95 (75–100) and a slightly lower mean ATT of 1.7 ± 1.2 mm [24]. However, most of the studies in the recent literature have a follow‐up of <5 years and only a small proportion of them evaluate the development of arthritis.

We presented the arthritic evolution according to the KL classification of patients undergoing ACLR in the two groups without finding any differences between the groups but nevertheless showing an evolution at the follow‐up; exclusion criteria can also influence our data. No comparative study of these two techniques describes the OA evolution.

Grassi et al. in a recent meta‐analysis with over 20 years of follow‐up after ACLR, but using different surgical techniques to our work, showed that signs of OA were reported in 73.3% of patients, while severe OA was reported in 12.8%. The operated knee had a relative risk of OA of 2.8 (*p* < 0.001) compared with the contralateral knee. Identified risk factors for long‐term OA were male sex, older age at surgery, delayed ACLR, meniscal or cartilage injury, BPTB autograft, lateral plasty, nonideal tunnel placement, residual laxity, higher postoperative activity and postoperative range of motion deficits [17].

The MOON knee group in an observational multicentric study showed that in young active patients, the 10‐year incidence of clinical radiographic OA after ACLR was 37% as defined by osteophytes and 23% as defined by joint space narrowing [14].

In a meta‐analysis, Cinque et al. demonstrated that increasing chronicity of the ACL tear prior to surgery and increasing mean patient age at surgery were associated with a higher likelihood of OA development [8].

We do not have the necessary data to calculate OA progression compared to the contralateral knee and compared to time since surgery.

The strengths of the study are the rigorous inclusion and exclusion criteria to try to make the two groups similar and avoid bias, not only in terms of age, sex and follow‐up but also by excluding associated procedures or patients with chondral damage or arthrosis KL > 3 or significant meniscal lesions requiring subtotal meniscectomy or sutures. In addition, only a few patients refused to take part in the trial. Nevertheless, this study has some weaknesses. Patients were enrolled for surgery in two different centres and have been evaluated by two different senior fellows, and this could potentially cause some bias in the results.

Another limitation of the study is that there is no mention of tibial slope in the exclusion criteria, as this analysis was not performed, and sports activity was not investigated.

## CONCLUSION

Our data show significant improvements in all clinical outcome measures along with excellent KT‐1000 arthrometer values and low clinical failure rates for both the SB all‐inside and modified DB techniques at a mean follow‐up of over 6 years. Furthermore, there are no differences in arthritic evolution between groups according to KL grade. The hypothesis of the study was confirmed because we did not find a significant difference between the two techniques. More long‐term follow‐up could be useful to understand if the bone preservation in the all‐inside technique could have some influence to better preserve the long‐term stability of the knee.

## AUTHOR CONTRIBUTIONS

**Raffaele Garofalo**: Conceptualisation; original draft preparation. **Francesco Fiore**: Data curation. **Giuseppe Danilo Cassano**: Writing—original draft preparation. **Lorenzo Moretti**: Writing—review and editing. **Giuseppe Solarino**: Supervision. All authors have read and agreed to the published version of the manuscript.

## CONFLICT OF INTEREST STATEMENT

The authors declare no conflicts of interest.

## ETHICS STATEMENT

The study was conducted according to the guidelines of the Declaration of Helsinki. Ethical clearance was obtained from our Local Ethical Committee (approval n. 6745), and all patients gave informed consent before enrolment in the study. Informed consent was obtained from all subjects involved in the study. Written informed consent has been obtained from the patient(s) to publish this paper if applicable.

## Data Availability

The data presented in this study are available on request from the corresponding author. The data are not publicly available due to privacy.
